# Two single arm trials of AKL-T01, a digital therapeutic for adolescents and adults with ADHD

**DOI:** 10.1038/s44184-024-00075-w

**Published:** 2024-06-19

**Authors:** Caitlin A. Stamatis, Deborah N. Farlow, Catherine Mercaldi, Minny Suh, Amanda Maple, Antonia Savarese, Ann Childress, Raun D. Melmed, Scott H. Kollins

**Affiliations:** 1 Akili Interactive Labs, Boston, MA USA; 2 Department of Preventive Medicine, Northwestern University Feinberg School of Medicine, Chicago, IL USA; 3 Center for Psychiatry and Behavioral Medicine, Las Vegas, NV USA; 4 Melmed Center/Cortica, Scottsdale, AZ USA; 5 Department of Psychiatry, Duke University School of Medicine, Durham, NC USA

**Keywords:** Attention, ADHD

## Abstract

Inattention symptoms represent a key driver of functional impairment in ADHD and often persist into adolescence and adulthood, underscoring a need for novel treatments targeting attentional control. We evaluated AKL-T01—a digital therapeutic that is FDA-cleared for children 8–12 y with ADHD—in adolescents and adults with ADHD in two independent single-arm trials: STARS-ADHD-Adolescent, a 4-week trial in adolescents 13–17 y (*n* = 162 enrolled), and STARS-ADHD-Adult, a 6-week trial in adults 18 and older (*n* = 221 enrolled). AKL-T01 was linked with improvements on the Test of Variables of Attention (TOVA^®^) Attention Comparison Score (ACS) of 2.6 (95% CI: 2.02, 3.26*; p* < 0.0001) in adolescents and 6.5 in adults (95% CI: 5.35, 7.57; *p* < 0.0001), along with improvements in secondary endpoints. 15 participants reported adverse device effects, all mild or moderate. Though limited by a single-arm design, results provide preliminary support for the safety and efficacy of AKL-T01 for adolescents and adults with ADHD.

## Introduction

Attention-deficit/hyperactivity disorder (ADHD) is one of the most common pediatric psychiatric conditions, and with the recognition that symptoms often persist into adulthood^1^, rates of treatment seeking for adult ADHD have increased significantly in recent years^2^. These trends are echoed in the popular press, with particular attention toward increased rates of adult ADHD diagnoses and medication shortages highlighting a need for improved access to care^3,4^. Current treatments for ADHD have demonstrated incontrovertible short-term benefits across core symptoms and associated impairments^5,6^, but have limitations. Pharmacological treatments such as stimulant medications are associated with well-documented side effects, and they may not produce optimal benefits to certain cognitive domains^7–9^. Nonpharmacological interventions (e.g., cognitive behavioral therapy) can be difficult to access, in part due to the limited number of qualified providers^10,11^. Moreover, since cognitive behavior therapy is not regulated, the quality and fidelity of the treatment in real world settings is not consistent with clinical trials supporting its efficacy. Adherence rates over time are generally low for existing standard of care treatment for ADHD, and longitudinal studies have shown that improvements are not maintained beyond 24–36 months^12,13^. While ADHD symptoms and functional impairment persist throughout adolescence and adulthood, medication use declines after age 11, and those who continue medication tend to do so inconsistently^14,15^.

Limitations of front-line treatments underscore a need for innovation in the development of efficacious and scalable interventions for ADHD. Given the central role of attention and related cognitive processes in ADHD pathophysiology^16^, interventions that specifically target these constructs in safe and effective ways are critical. This is especially true as patients with ADHD move into adolescence and adulthood, with inattention becoming the predominant symptom domain endorsed in these age groups^17^. Computerized cognitive training (CCT) programs have been associated with improvements to working memory and executive functioning in ADHD populations^18,19^. Meta-analyses suggest non-significant evidence of far transfer (i.e., to impact ADHD symptoms; academic impairment) with CCT, and that evidence weakens with unblinded raters^19^. However, multi-component training models (i.e., CCT targeting multiple cognitive processes, and not just working memory) show promise, likely due to the heterogeneity of neuropsychological deficits in ADHD^19^. One such treatment, AKL-T01, is a novel digital therapeutic currently authorized by the US Food and Drug Administration (FDA) as EndeavorRx^®^ (DEN200026)^20^. EndeavorRx is indicated to improve attentional functioning in children ages 8 to 12 years old with primarily inattentive or combined-presentation ADHD and demonstrated attentional impairment^21^. AKL-T01 is deployed on mobile devices (tablets and phones) and targets a range of processes implicated in attentional control through cognitive tasks in a videogame-based platform, using a staircase algorithm that personalizes level of difficulty to each individual.

Two pivotal clinical trials support the safety and efficacy of AKL-T01 for targeting inattention and clinical functioning in pediatric ADHD samples. The first, Software Treatment for Actively Reducing Severity of ADHD (STARS-ADHD), was a multicenter, randomized, double-blind, active-controlled study comparing AKL-T01 to a digital control in children 8–12 years old, diagnosed with ADHD, with impairment in objective attention function (as indicated by the Test of Variables of Attention [TOVA]-Attention Performance Index [API] ≤ −1.8), and not taking ADHD medication^22^. The AKL-T01 group demonstrated significantly greater improvements to attention on the TOVA-API compared to the active control group^23^. The second study, STARS-ADHD-Adjunctive, was a single-arm trial of AKL-T01 in children ages 8–14, both on and off stimulant medication. Comparable improvements were seen across the medication and non-medication cohorts in ADHD-related impairment and symptoms^24^. AKL-T01 demonstrated a favorable safety profile, with no serious adverse device effects and a relatively small number of mild to moderate adverse device events (ADEs) in both studies. Together, these trials suggest that AKL-T01 is safe and effective for children with ADHD, and that benefits to attentional functioning and functional impairment can be derived even among those actively participating in gold-standard treatment.

Given the rising demand for adult ADHD treatment^2^, the centrality of inattentive symptoms as patients age^17^, and the need for expanded access to evidence-based interventions for ADHD, an important question is whether similar benefits with AKL-T01 are observed in adolescents and adults with ADHD as in the pediatric population. In the present study, we evaluated the safety and efficacy of AKL-T01 for improving objective attention and ADHD-related symptoms in two independent single-arm clinical trials with no comparison group, one in adolescents ages 13–17 (STARS-ADHD-Adolescent), and the other in adults ages 18 and older (STARS-ADHD-Adult). Since both of these studies were explicitly designed to demonstrate substantial equivalence as part of regulatory submissions, they did not include a comparison arm, as supported by regulatory guidance in CFR 806.7(c)(2). Additional information supporting the single-arm trial design, including an extensive discussion of placebo effects and sensitivity analyses, is contained in the Supplementary Materials: Single-Arm Trial.

## Results

### Participants

Table 1 contains detailed demographic characteristics for each trial.

**Table 1:** Baseline characteristics by study population

	Adolescent			Adult		
Characteristic	Safety population (*N* = 162)	Efficacy population (*N* = 146)	Per protocol population (*N* = 74)	Safety population (*N* = 221)	Efficacy population (*N* = 153)	Per protocol population (*N* = 96)
Age at baseline (years)						
*n*	162	146	74	221	153	96
Mean (SD)	14.4 (1.23)	14.3 (1.26)	14.2 (1.17)	39.0 (12.69)	39.9 (12.84)	40.6 (12.37)
Min, max	13, 17	13, 17	13, 17	18, 76	18, 76	19, 76
Age at ADHD symptom onset (years)						
*n*	160	144	74	191	133	83
Mean (SD)	5.7 (2.82)	5.8 (2.89)	5.7 (2.95)	8.2(5.03)	8.0 (4.55)	8.1 (4.17)
Min, max	1, 15	1, 15	2, 15	3,42	3,36	4,36
Age at ADHD diagnosis (years)						
*n*	158	142	72	216	148	93
Mean (SD)	8.7 (3.33)	8.7 (3.38)	8.7 (3.48)	27.2 (15.31)	28.6 (15.31)	29.5 (15.95)
Min, Max	3, 17	3, 17	3, 15	3,69	4,69	4,69
Sex [n (%)]						
*n*	162	146	74	221	153	96
Female	66 (40.7%)	60 (41.1%)	33 (44.6%)	147 (66.5%)	107 (69.9%)	65 (67.7%)
Male	96 (59.3%)	86 (58.9%)	41 (55.4%)	74 (33.5%)	46 (30.1%)	31 (32.3%)
Race [n (%)]						
*n*	162	146	74	221	153	96
American Indian or Alaska Native	3 (1.9%)	3 (2.1%)	0	2 (0.9%)	1 (0.7%)	1 (1.0%)
Asian	1 (0.6%)	1 (0.7%)	0	8 (3.6)	7 (4.6%0	7 (7.3%)
Black or African American	17 (10.5%)	15 (10.3%)	7 (9.5%)	26 (11.8%)	15 (9.8%)	6 (6.3%)
White	125 (77.2%)	113 (77.4%)	61 (82.4%)	166 (75.1%)	117 (76.5%)	74 (77.1%)
Multiple	13 (8.0%)	11 (7.5%)	4 (5.4%)	11 (5.0%)	7 (4.6%)	6 (6.3%)
Other	3 (1.9%)	3 (2.1%)	2 (2.7%)	6 (2.7%)	5 (3.3%)	2 (2.1%)
Ethnicity [n (%)]						
*n*	162	146	74	221	153	96
Hispanic or Latino	29 (17.9%)	26 (17.8%)	17 (23.0%)	31 (83.7%)	23 (15%)	11 (11.5%)
Not Hispanic or Latino	132 (81.5%)	119 (81.5%)	56 (75.7%)	185 (83.7%)	126 (82.4%)	83 (86.5%)
Not reported	1 (0.6%)	1 (0.7%)	1 (1.4%)	5 (2.3%)	4 (2.6%)	2 (2.1%)

*ADHD* Attention Deficit Hyperactivity Disorder, *max* maximum, *min* minimum.

#### Adolescents

**Fig. 1:**
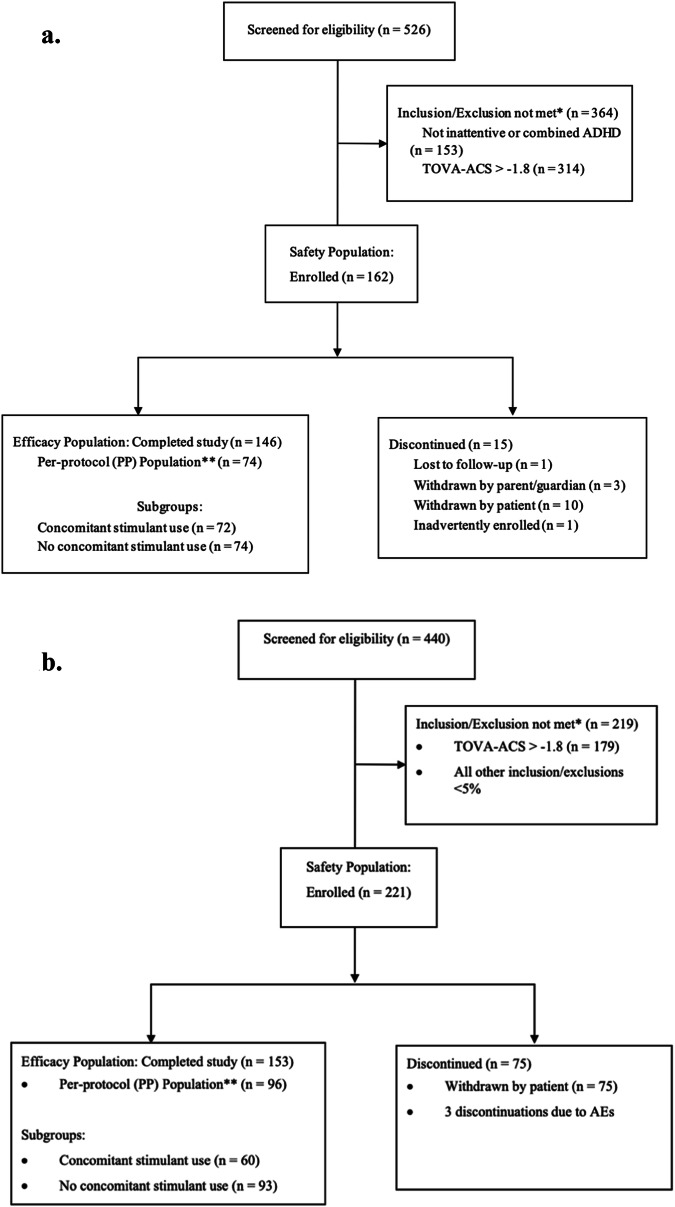
Consort Diagrams. **a** Trial Consort Flow Diagram for STARS-ADHD-Adolescent Trial. **b** Trial Consort Flow Diagram for STARS-ADHD-Adult Trial. *Participants could have more than one reason for exclusion recorded. The two most frequently failed criteria are reported. **At least 60% of prescribed missions, equivalent to 72 of 120 missions and whose exit visit occurred between Day 22 and Day 34.

Of 526 participants screened, 162 were enrolled between July 29, 2021 and September 1, 2022 (Fig. 1a) from across 13 sites (see Supplementary Materials: Poolability for post-hoc analyses supporting pooling data across sites). The most common reason for screen failure was a baseline score > −1.8 on the TOVA Attention Comparison Score (ACS; *n* = 314). All 162 enrolled were included in the Safety Population, 146 were included in the Efficacy Population, and 74 were included in the Per Protocol Population (see Methods for definitions). Fifteen participants (9.3%) discontinued the study. In the Efficacy Population, participants were predominantly male (58.9%), White (77.4%) and non-Hispanic/Latino (81.5%), with a mean age of 14.3 years (SD = 1.26). Approximately half (49.3%) reported current stimulant use, and 18.5% reported concurrent psychosocial treatment.

#### Adults

Of 440 participants screened, 221 participants were enrolled between November 29, 2021 and December 2, 2022 (Fig. 1b) from across 13 sites (see Supplementary Materials: Poolability for post-hoc analyses supporting pooling data across sites). The most common reason for screen failure was a baseline TOVA-ACS > −1.8 (*n* = 179). All 221 enrolled were included in the Safety Population, 153 were included in the Efficacy Population, and 96 were included in the Per Protocol Population. Seventy-five participants (34%) discontinued the study. In the Efficacy Population, participants were predominantly female (70%), White (77%) and non-Hispanic/Latino (85%), with a mean age of 39.9 years (SD = 12.84). Current stimulant use was reported by 39.2% of participants.

### Compliance

#### Adolescents

Mean overall compliance in the Efficacy Population was 72.4%, with an average (SD) of 288 (169) minutes of treatment exposure over 84 (47) missions. Over half (57.5%) of participants in the Efficacy Population met the Per Protocol definition of a minimum of 60% treatment compliance.

#### Adults

Mean overall compliance in the Efficacy Population was 81.1%, with an average (SD) of 530 (320) minutes of treatment exposure over 146 (86) missions. The majority (67.3%) of participants in the Efficacy Population met the Per Protocol definition of a minimum of 60% treatment compliance.

Across both studies, mean compliance trended downward slightly across study weeks, and this trend was consistent for all analysis populations and subgroups. See Supplementary Table 5 and Supplementary Table 6 for percent compliance by week.

### Efficacy

#### Adolescents

TOVA-ACS significantly improved from baseline to study day 28, with a mean increase of 2.64 (*SD* = 3.80), 95% CI [2.02, 3.26], *t*(145) = 8.40, *p*  <  0.0001, *d* [95% CI] = 0.70 [0.54, 0.87] (Table 2). Post-hoc sensitivity analyses using Wilcoxon Signed Rank tests of change in TOVA-ACS from baseline to exit yielded similar results (*p* < 0.0001). Both secondary ADHD-Rating Scale-5 (ADHD-RS-5) endpoints also showed significant improvement from baseline to day 28: inattention subscale *M* change = −2.99 (*SD* = 5.38), 95% CI [−3.87, −2.10], *t*(144) = −6.69, *p* <  0.0001, *d* [95% CI] = 0.59 [0.41, 0.76]; total score *M* change = −4.59 (*SD* = 8.14), 95% CI [−5.93, −3.25], *t*(143) = −6.77, *p*  <  0.0001, *d* [95% CI] = 0.50 [0.35, 0.65] (Table 3). There were no significant differences in any study outcomes between the subgroups who were or were not taking concomitant stimulant medication for TOVA-ACS (*t*[136.42] = −1.10, *p* = 0.272), ADHD-RS-5 inattention (*t*[141.77] = −0.28, *p* = 0.781), or ADHD-RS-5 total score (*t*[137.75] = 0.63, *p* = 0.528).

**Table 2:** Primary Efficacy Endpoint – TOVA-ACS Change from Baseline to Day 28 or Day 42 for Efficacy Population in Adolescents (*N*  =  146) and Adults (*N* = 153)

	Adolescent			Adult		
Statistic	Baseline	Day 28	Change	Baseline	Day 42	Change
Mean (SD)	−5.45 (3.75)	−2.81 (4.28)	2.64 (3.80)	−8.74 (7.53)	−2.28 (4.92)	6.46 (6.95)
Median	−4.27	−2.40	2.42	−6.52	−1.19	4.86
Interquartile range	−7.32, −2.77	−5.25, 0.00	−0.35, 5.57	−9.98, −3.82	−5.51, −1.55	2.71, 7.93
Min, max	−24.23, −1.81	−16.01, 5.52	−5.26, 18.44	−41.53, −1.79	−16.52, 5.76	−3.63, 40.35
2-sided 95% CI^a^			2.02, 3.26			5.35, 7.57
*P*-value^b^			< 0.0001			<0.0001

*max* maximum, *min* minimum.

^a^ Positive change indicates improvement. Efficacy is concluded if the lower bound of the CI is > 0.

^b^ From a one-sample t-test.

**Table 3:** Secondary Efficacy Endpoints – ADHD RS Change from Baseline to Day 28 or 42 for Efficacy Population in Adolescent (*N*  =  146) and Adult (*N* = 153) Samples

Parameter	Statistic	Baseline	Day 28 (Adolescent) or 42 (Adult)	Change from baseline
Adolescent ADHD-RS-5 Inattention subscale	*n*	145	146	145
	Mean (SD)	18.91 (5.09)	15.98 (5.61)	−2.99 (5.38)
	Median	20.0	16.0	−3.0
	Interquartile range	16.0, 23.0	12.0, 21.0	−6.0, 0.0
	Min, max	2, 27	2, 27	−20, 10
	2-sided 95% CI^a^			−3.87, −2.10
	*P*-value^b^			<0.0001
Adolescent ADHD-RS-5 Total score	*n*	144	146	144
	Mean (SD)	31.5 (9.17)	27.0 (9.95)	−4.59 (8.14)
	Median	31.0	26.0	−4.5
	Interquartile range	25.5, 38.0	21.0, 35.0	−10.0, 1.0
	Min, max	8, 52	3, 51	−32, 17
	2-sided 95% CI^a^			−5.93, −3.25
	*P*-value^b^			< 0.0001
Adult ADHD-RS-IV Inattention subscale	*n*	153	153	153
	Mean (SD)	21.8 (3.26)	16.7 (4.98)	−5.1 (4.78)
	Median	22.0	17.0	−5.0
	Interquartile range	20.0, 24.0	13.0, 20.0	−8.0, −1.0
	Min, max	12, 27	5, 27	−20, 5
	2-sided 95% CI^a^			−5.86, −4.34
	*P*-value^b^			<0.0001
Adult ADHD-RS-IV Total score	*n*	153	153	153
	Mean (SD)	38.2 (7.41)	30.0 (9.40)	−8.3 (7.74)
	Median	38.0	29.0	−8.0
	Interquartile range	33.0, 43.0	24.0, 36.0	−13.0, −3.0
	Min, max	24, 54	9,5 3	−32, 9
	2-sided 95% CI^a^			−9.51, −7.04
	*P*-value^b^			<0.0001

*ADHD RS* Attention Deficit Hyperactivity Disorder Rating Scale, *max* maximum, *min* minimum.

^a^ Negative change indicates improvement. Efficacy was concluded if the upper bound of the CI was < 0.

^b^ From a one-sample t-test.

#### Adults

TOVA-ACS significantly improved from baseline to study day 42, with a mean increase of 6.46 (*SD* = 6.95), 95% CI [5.35, 7.57], *t*(152) = 11.49, *p* <0.0001, *d* [95% CI] = 0.86 [0.71, 1.01] (Table 2). Post-hoc sensitivity analyses using Wilcoxon Signed Rank tests of change in TOVA-ACS from baseline to exit yielded similar results (*p* < 0.0001). Both secondary ADHD-Rating Scale-IV (ADHD-RS-IV) endpoints also showed significant improvement from baseline to day 42: inattention subscale *M* change = −5.10 (*SD* = 4.78), 95% CI [−5.86, −4.34], *t*(152) = −13.20, *p* < 0.0001, *d* [95% CI] = 1.56 [1.33, 1.80]; total score *M* change = −8.27 (*SD* = 7.74), 95% CI [−9.51, −7.04], *t*(152) = −13.23, *p* < 0.0001, *d* [95% CI] = 1.12 [0.95, 1.28]. Similarly, adults reported clinically significant improvements from baseline to day 42 on the Conners’ Adult ADHD Rating Scales (CAARS) ADHD index, *M* change = −4.30 (*SD* = 9.18), 95% CI [−5.77, −2.83], *t*(152) = −5.79, *p* <0.0001, *d* [95% CI] = 0.30 [0.15, 0.45], and in quality of life on the Adult ADHD Quality of Life (AAQoL), *M* change = 7.84 (*SD* = 13.75), 95% CI [5.64, 10.04], *t*(152) = 7.05, *p* <0.0001, *d* [95% CI] = 0.55 [0.40, 0.71]. There were no significant differences between the subgroups who were versus were not taking concomitant stimulant medication in change on the TOVA-ACS (*t*[104.21] = −1.21, *p* = 0.231), ADHD-RS-IV inattention (*t*[107.56] = 1.61, *p* = 0.111), ADHD-RS-IV total score (*t*[105.76] = 1.09, *p* = 0.277), or CAARS ADHD index (*t*[123.83] = −1.51, *p* = 0.133). Those in the stimulant group reported greater improvements in AAQoL from baseline to day 42 (*M* change = 10.66) relative to those not taking stimulants (*M* change = 6.02; *t*[120.82] = −2.03, *p* = 0.044), though this difference was non-significant after correction for multiple comparisons.

### Responder analysis

#### Adolescents

In the Efficacy Population, 61.0% were responders based on a 1.4-point improvement on TOVA-ACS (60.8% in the Per Protocol Population), 24.7% were responders based on a TOVA-ACS ≥  0 at exit visit (27.0% in the Per Protocol Population), and 27.1% were responders based on a ≥  30% improvement in ADHD RS-5 total score (27.0% in the Per Protocol Population; Table 4).

**Table 4:** Exploratory responder analysis for efficacy population in adolescent (*N*  =  146) and adult (*N* = 153) trials

Study	Parameter	n/N (%)
Adolescent	TOVA-ACS: 1.4-point improvement from baseline to day 28	89/146 (61.0%)
	TOVA-ACS: Score at exit visit ≥ 0	36/146 (24.7%)
	ADHD RS-5 Total Score: ≥ 30% improvement from baseline to day 28	39/144 (27.1%)
Adult	TOVA-ACS: 1.4-point improvement from baseline to day 42	127/153 (83.0%)
	TOVA-ACS: Score at exit visit ≥ 0	56/153 (36.6%)
	ADHD RS-IV Total Score: ≥ 30% improvement from baseline to day 42	50/153 (32.7%)
	AAQoL: ≥ 8 point improvement from baseline to day 42	70/153 (45.8%)

*ADHD RS-5* Attention Deficit Hyperactivity Disorder Rating Scale-5, *TOVA-ACS* Test of Variables of Attention-Attention Comparison Score, *ADHD RS-IV* Attention Deficit Hyperactivity Disorder Rating Scale-IV.

#### Adults

In the Efficacy Population, 83.0% were responders based on a 1.4-point improvement on the TOVA-ACS (84.4% in the Per Protocol Population), 36.6% were responders based on a TOVA-ACS ≥  0 at exit visit (41.7% in the Per Protocol Population), 27.1% were responders based on a ≥  30% improvement in ADHD RS-IV total score (34.4% in the Per Protocol Population), and 32.7% were responders based on a ≥ 8-point improvement on the AAQoL (44.8% in the Per Protocol Population).

### Safety results

#### Adolescents

4 participants (2.5%) experienced a treatment-emergent ADE (TE-ADE; Table 5), most commonly decreased frustration tolerance (1.9%) and headache (0.6%). No TE-ADEs were serious or resulted in study discontinuation, and no events were unanticipated as defined by the protocol.

**Table 5:** Summary of TE-ADEs by SOC and PT for Safety Population (*N* = 162) Adolescent

	All Subjects (*N* = 162)	
	n (%)	Events
*Any TE-ADE*	*4 (2.5%)*	*4*
Nervous system disorders	1 (0.6%)	1
Headache	1 (0.6%)	1
Psychiatric disorders	3 (1.9%)	3
Frustration tolerance decreased	3 (1.9%)	3

*TE-ADE* treatment-emergent adverse device effect. *SOC* System Organ Class (categories), *PT* Preferred Term (indented; subcategories).

#### Adults

11 participants (5%) experienced a TE-ADE (Table 6), most commonly nausea (1.8%) and headache (1.4%). No TE-ADEs were serious. 3 (1.4%) were unanticipated and resulted in study discontinuation; these TE-ADEs included headache (1 participant) and nausea (2 participants), and all were considered recovered/resolved after study treatment discontinuation.

**Table 6:** Summary of TE-ADEs by SOC and PT for Safety Population (*N* = 221) Adult

	All Subjects (*N* = 221)	
	*n* (%)	Events
*Any TE-ADE*	*13 (5.8%)*	*13*
Cardiac disorders	1 (0.5%)	1
Dizziness	1 (0.5%)	1
Gastrointestinal disorders	4 (1.8%)	4
Nausea	4 (1.8%)	4
General disorders and administration site conditions	1 (0.5%)	1
Fatigue	1 (0.5%)	1
Musculoskeletal and connective tissue disorders	1 (0.5%)	1
Arthritis	1 (0.5%)	1
Nervous system disorders	4 (1.8%)	4
Headache	3 (1.4%)	3
Somnolence	1 (0.5%)	1
Psychiatric disorders	2 (0.9%)	2
Frustration tolerance decreased	2 (0.9%)	2

*TE-ADE* treatment-emergent adverse device effect. *SOC* System Organ Class (categories), *PT* Preferred Term (indented; subcategories).

## Discussion

Across the two clinical trials described, we evaluated the use of AKL-T01—which had previously been demonstrated effective in children ages 8–12 with ADHD^23,24^—for adolescents and adults with ADHD. Results provide preliminary support for the efficacy of AKL-T01; while the studies cannot be directly compared to prior work in younger children due to methodological differences, the magnitude of the effects of AKL-T01 in adolescents and adults was generally medium to large. Further, AKL-T01 was associated with benefits to ADHD-related symptoms across age groups and improvements to quality of life among adult participants, suggesting that treating inattention via video-game-based cognitive control training may translate to real-world benefits.

**Fig. 2:**
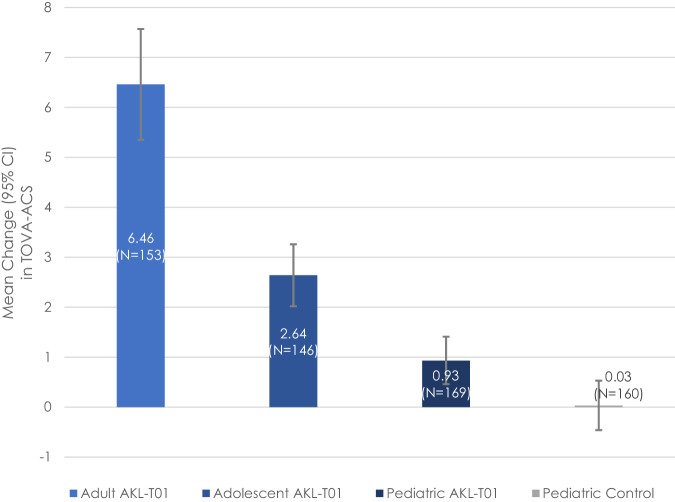
TOVA-ACS change across adult and adolescent trials described here, and in prior pediatric trial. Pediatric data is from previously published randomized controlled trial of AKL-T01 against an active digital control^23^.

In the context of prior pediatric studies^23,24^, our results in adolescents and adults suggest that AKL-T01’s benefits to attentional functioning extend across the lifespan. Across both trials presented, participants demonstrated significant increases in TOVA-ACS. The TOVA is an objective measure of attentional functioning with low likelihood of expectation of benefit bias (i.e., placebo effect)^22^; supporting this, no placebo effect for TOVA was observed in the pediatric STARS trial that did include a control condition (see Supplementary Materials: Single-Arm Trial for further discussion of placebo effects). Notably, the magnitude of TOVA-ACS change became larger across age groups (adolescent *M* change = 2.64; adult *M* change = 6.46; Fig. 2). As previously stated, differences in trial methodology temper the ability to directly compare TOVA-ACS effects from these two trials to the prior pediatric RCT. However, considering within-treatment-group effect sizes, the greater improvements in attentional functioning observed in adolescents (nearly 3× higher) and adults (nearly 7x higher) relative to children may stem from differences across the populations in motivation and clinical characteristics. In terms of clinical characteristics, adults with ADHD are more likely to report inattentive symptoms^17,25^, which reflect the primary symptom domain targeted by AKL-T01. In contrast, medications targeting broad ADHD symptoms show similar effect sizes in children, adolescents, and adults^9^. Regarding motivation, whereas pediatric engagement with AKL-T01 is typically influenced by a caregiver, adults generally self-select into treatment and are therefore more likely to benefit from self-efficacy, a pivotal value in health behavior change^26^.

Along with objective attentional functioning, AKL-T01 was associated with benefits to ADHD symptoms across age groups, as measured by the ADHD-RS. The ADHD-RS is a clinician-administered measure that is widely used to assess ADHD symptoms in clinical trials with children, adolescents, and adults^14,15,27^. Relative to prior pediatric trials^23,24^, we observed ADHD-RS changes of larger magnitude in the adult data and slightly smaller changes in the adolescent data, though comparisons must be considered in the context of trial methodology differences. Given that participants in the adolescent trial had baseline scores indicating fewer symptoms at study start than in the STARS-ADHD pediatric trial, results suggest a similar relative effect of AKL-T01 on ADHD symptoms across studies. Additionally, in prespecified responder analyses, a comparable percentage of participants in each study (27%) achieved a 30% improvement in ADHD-RS total score, which is widely accepted as a minimal clinically important difference^28^. Given the evidence in the adult study that AKL-T01 was also associated with benefits to quality of life on the AAQoL, it will be important to similarly evaluate impacts to daily functioning in adolescents, which were not evaluated in the present report.

A critical finding that emerged in the prior pediatric trials of AKL-T01^23,24^, and which is echoed in the adolescent and adult trials presented here, is that AKL-T01 appears to have comparable effects regardless of whether the patient is concomitantly treated with stimulant medication. Clinical guidelines support medication and cognitive-behavioral therapy as first-line interventions for the treatment of ADHD^6^. Our finding that patients taking stimulant medication seemed to benefit from AKL-T01 beyond their existing medication regimen aligns with evidence that multimodal treatment yields superior outcomes for ADHD symptoms and functional impairment^29^. The improvements across groups to attention and ADHD symptoms suggests that AKL-T01 is beneficial for patients with ADHD whose inattention is not currently well-controlled with stimulants, as well as those who do not include stimulant medication as part of their current ADHD treatment. It will be important to continue to evaluate potential differences in AKL-T01 treatment outcomes according to concomitant medication status with larger studies explicitly powered to detect potential subgroup differences, and geared toward understanding how the timing of stimulant medication use relative to AKL-T01 engagement may influence results.

Strengths and limitations of the present study point to directions for future research. Strengths include relatively large samples, multisite trials, and a consistency of design across both studies that allowed for comparisons across the lifespan. One limitation of both trials presented here is the single-arm design, which tempers the strength of conclusions about clinical efficacy. The lack of change in TOVA-ACS in the active control group in the previously described pediatric RCT provides indirect evidence against a placebo effect (see also Fig. 2); whether the same holds true for adults and adolescents remains unknown (though see Supplementary Materials: Single-Arm Trial for justification of the single-arm trial design). While the single-arm trials cannot be directly compared to the pediatric RCT, the general magnitude of effects in adolescents and adults, which exceeded the previously reported effect of AKL-T01 in children by 2- to 7-fold, supports a beneficial impact of the intervention beyond regression to the mean or placebo effect. Given that rating scales may be more subject to placebo effects than the TOVA (e.g., if participants previously heard about the FDA clearance of EndeavorRx and had expectancy bias), it will be important for future studies to consider the impact of AKL-T01 on ADHD symptoms and quality of life relative to an active control. Another consideration is dropout, which was higher in the adult study than in the adolescent trial or prior pediatric trials, though comparable to dropout rates in recent ADHD medication trials^30,31^. Future studies should consider possible reasons for differential dropout rates (e.g., the longer duration of treatment in the adult study; parental accountability among adolescents). Additional studies to investigate longer term benefits of AKL-T01 in adults and adolescents are needed to determine whether AKL-T01 has lasting effects on attention and related processes. It will also be important to test the generalizability of the intervention’s effects to adults without TOVA-ACS impairment at baseline, and to understand predictors of response vs. non-response to treatment. Finally, future studies are needed to evaluate the minimum effective dose of AKL-T01, particularly to see if regimens involving a lower time commitment than the recommended 25 minutes per day, 5 days a week can lead to comparable outcomes.

In two independent trials in adolescents and adults with ADHD, AKL-T01 demonstrated a favorable safety profile and consistent clinical benefit, resulting in significant improvement in attention functioning, ADHD symptoms, and, in adults, quality of life. The magnitude of response was comparable to or larger than that of previous studies of this digital therapeutic, with particularly strong benefits in adults. Given the increased rates of treatment-seeking in adults with ADHD^2^, the significant barriers to accessing empirically-supported treatments^10,11^, and the centrality of inattentive symptoms as ADHD patients develop into adulthood^28^, AKL-T01 holds promise as a scalable, targeted treatment for inattention in adolescent and adult ADHD with evident impact on real-world symptoms. Consistent evidence across multiple trials supports the value of AKL-T01 for patients currently taking stimulant medications, in line with clinical guidelines supporting a multimodal treatment regimen that incorporates behavioral, pharmacological, and now digital interventions.

## Methods

### Overview

The STARS-ADHD-Adolescent trial (NCT04897074) was a multicenter, single-arm, open-label study to evaluate objective attention functioning and ADHD symptoms in adolescents aged 13 to 17 years 10 months old, with a diagnosis of ADHD (combined or inattentive presentation), stably on or off ADHD medication, after 4 weeks of AKL-T01. The STARS-ADHD-Adult trial (NCT05183919) was a multicenter, single-arm, open-label study to evaluate objective attention functioning and ADHD symptoms and impairments in adults 18 years and older diagnosed with combined or inattentive ADHD, stably on or off ADHD medication, after 6 weeks of AKL-T01.

The trials were conducted in compliance with the Institutional Review Board (IRB) regulations stated in Title 21 of the US Code of Federal Regulations (CFR), Part 56, Good Clinical Practice (GCP) regulations and guidelines, and all applicable local regulations. The trials were approved by each site’s institutional review board (WIRB-Copernicus Group). Written informed consent (and assent for adolescents) was obtained from all participants and (for adolescents) their parent or legally authorized representative, respectively, and as appropriate given the participants’ ages. IRB-approved forms containing a detailed description of the study treatment, study procedures, and risks were provided to participants and (for the adolescent study) caregivers.

### Study design

The STARS-ADHD-Adolescent study was designed to enroll up to 165 participants from up to 20 US-based sites. Study participation included a screening visit, a baseline visit (on-site; day 1), the 28-day treatment phase (at home; days 2 to 27), and an exit visit on day 28 ( ± 3 days; on-site). AKL-T01 treatment involved playing 6 to 8 missions of the game per day, for at least 5 days per week, for 4 consecutive weeks.

The STARS-ADHD-Adult study was initially designed to enroll up to 325 participants, with a minimum sample size of 301. An adaptive study design was adopted to enroll participants until the Standard Error (SE) of mean TOVA‑ACS change was ≤ 0.277, the threshold required to maintain 90% power to detect changes in the primary endpoint. However, the adult trial was discontinued prior to reaching the prespecified enrollment numbers for two primary reasons unrelated to safety or efficacy: First, the adolescent trial had demonstrated robust and meaningful effects that were larger than anticipated; second, projections based on enrollment rates indicated that the trial may not have reached completion with the target sample size until 2024. Although the stopping rule based on the target SE was not achieved, prespecified efficacy analyses yielded *p*-values <  0.000001 for primary and secondary outcomes necessary for making efficacy-related decisions. Study participation included a screening visit, a baseline visit (on site; study day 1), the 42‑day treatment phase (at home; days 2 to 42), and an exit visit on day 42 ( ± 3 days; on site). AKL-T01 treatment involved playing 6 to 8 missions per day, for at least 5 days per week, for 6 consecutive weeks, consistent with prior studies of this technology in adults^32^.

### Participants

For STARS-ADHD-Adolescent, primary inclusion criteria were ages between 13 and 17 years, 10 months inclusive at the time of consent, and a diagnosis of ADHD combined or inattentive presentation, according to Diagnostic and Statistical Manual of Mental Disorders, Fifth Edition (DSM-5)^5^ as confirmed by MINI-Kid version 7.0.2^33^. Participants were also required to have a demonstrated attentional impairment defined by TOVA-ACS ≤ −1.8. All participants had an IQ score ≥ 80 (as assessed by KBIT-II)^34,35^ and the absence of any medical condition that could impact study participation or potentially confound study assessments. Full study inclusion and exclusion criteria are available in Supplementary Table 7.

Main inclusion criteria for STARS-ADHD-Adult were adults 18 years and older with a confirmed diagnosis of ADHD combined or inattentive presentation, according to DSM-5^5^ as confirmed by the MINI for Attention-Deficit/Hyperactivity Disorders Studies (Adult) version 7.0.2^36^. Participants were also required to have a demonstrated attentional impairment defined by TOVA‑ACS  ≤  −1.8 and ADHD RS‑IV ≥  24. Full study inclusion and exclusion criteria are available in Supplementary Table 8.

### Procedures

At baseline, participants received an iPad Mini with AKL-T01 software and completed training to learn proper device and software usage.

At the screening and exit visits, participants were assessed via validated measures of cognitive functioning (TOVA) and clinical symptoms (see “Outcomes”). Participants were instructed to avoid caffeine for 4–6 hours prior to the visit. At the investigator’s discretion, participants may also have been instructed to delay that day’s ADHD medication until after completion of the TOVA at both screening and exit visits to avoid unintentional impact of ADHD medication on TOVA performance^37^.

Participants completed AKL-T01 treatment at home in combination with their previously established stable ADHD treatment regimen (which could include stimulant medications, other psychoactive medications, and nonpharmacological therapies), and keep their previously established treatment stable for 4+ weeks prior to enrollment and throughout the study. Recommended treatment with AKL-T01 involved playing 6 to 8 missions per day (approximately 25 min) for at least 5 days per week for 4 weeks (adolescents) or 6 weeks (adults). Compliance was monitored electronically, and the software generated automatic reminders to play. The treatment automatically locked after the allocated maximum number of daily missions, and no further play was permitted until the next calendar day.

Any adverse device events (ADEs) occurring during any phase of the study judged by the principal investigator (PI) to be related to the intervention were recorded. If sites recorded mid-study discontinuation, they were required to indicate whether the discontinuation was due to an ADE or something else; if it was an ADE, then an ADE report was required. During the post-treatment visit, sites systematically collected data on whether participants reported any ADEs during the study period and, if so, completed an ADE report.

### Intervention

AKL-T01 is a digital therapeutic built using a proprietary algorithm (Selective Stimulus Management Engine [SSME™]) designed to train interference management at an adaptive and personalized high degree of difficulty. Interference was instantiated through a video game-based interface displaying two tasks done in parallel (multitasking): a perceptual discrimination targeting task in which users responded to the stimulus targets and ignored the distractors (similar to a Go-No-Go task), and a sensory motor navigation task in which users continuously adjusted their location to interact with or avoid positional targets. Performance in each task was assessed during single and multitask conditions. As users proceeded through the treatment, periodic recalibration occurred to maintain an optimal difficulty level.

### Outcomes

#### Primary endpoints

All efficacy outcomes were prespecified unless otherwise indicated. The primary endpoint of both studies was change (study day 1 to study day 28 [adolescent] or day 42 [adult]) on the TOVA-ACS, which is a composite score of objective attentional functioning, with norms relative to both ADHD and non-clinical samples. The clinical cutoff on the ACS is zero, with scores below zero observed in samples with ADHD and scores above zero typical of non-clinical samples. Positive changes in the ACS indicate improvement in objective attentional functioning^22^.

#### Secondary endpoints [adolescent]

Secondary endpoints of the STARS-ADHD-Adolescent study were change (study day 1 to study day 28) in the ADHD RS-5 inattention scale and total scores. The ADHD RS-5 is a parent-reported, clinician-administered assessment of the child’s frequency of ADHD symptoms, consisting of 18 items rated on a Likert scale ranging from 0 (never or rarely) to 3 (very often). The inattention scale score is the sum of the 9 items that comprise the inattention scale, and the total score is the sum of all 18 items^38^. We also examined responder rates, defined in three ways: (1) a 1.4-point improvement on TOVA-ACS from baseline after 4 weeks of AKL-T01 [Note that in the original adolescent study protocol, a TOVA-ACS change greater than or equal to 1.0 was prespecified as indicating a responder; however, we revised the criterion post-hoc to a more stringent TOVA-ACS change of 1.4 to be consistent with the prespecified definition of a responder as TOVA change equal to or greater than 1.4 points in the original pediatric STARS RCT.], (2) TOVA-ACS ≥  0 at exit visit (defined post-hoc), and (3) ≥  30% improvement in ADHD RS-5 total score from baseline after 4 weeks of AKL-T01.

#### Secondary endpoints [adult]

Secondary endpoints of the STARS-ADHD-Adult study were change (study day 1 to study day 42) in the clinician-administered ADHD RS-IV with Adult Prompts inattention subscale and total scores after 6 weeks of treatment with AKL-T01. We also examined change in the self-reported Adult ADHD Quality of Life (AAQoL)^39^, change in the Conner’s Adult ADHD Rating Scale–Self-report: Short Version (CAARS-S:S)^40^, and responder rates, defined in four ways: (1) a 1.4-point improvement on TOVA-ACS from baseline after 6 weeks of AKL-T01, (2) TOVA-ACS ≥  0 at exit visit (defined post-hoc), (3) ≥  30% improvement in ADHD RS-IV total score from baseline after 6 weeks of AKL-T01, and (4) AAQoL ≥ 8 point improvement from baseline after 6 weeks of AKL-T01.

#### Compliance

Percent compliance was derived as the number of completed missions divided by the expected minimum number of completed missions multiplied by 100. The minimum expected number of completed missions was 30/week, or 120 total in the adolescent study and 180 total in the adult study. Percent compliance could greater than 100 because the expected number of completed missions was based on 6 missions/day, 5 days/week, but participants were given the opportunity to complete up to 8 missions/day and play up to 7 days/week.

### Statistical analysis

All analyses were performed according to a prespecified analysis plan, unless indicated as post hoc. All analyses were conducted using a complete case analysis; no missing data were imputed.

#### Power analysis

Sample size was calculated based on a zero-change from baseline in TOVA-ACS against the alternative of a positive change from baseline in TOVA-ACS with a one-sample t-test using EAST^©^ version 6.5. For the adolescent trial, it was assumed that the TOVA-ACS SD (σ) in this population would be as large as observed with the pediatric population in the STARS-ADHD trial (SD  =  3.2). The significance level was defined by a one-sided α at 0.025 and achievement of a minimum power of 90%. Assuming a previously observed control-corrected effect size of µ = 0.9 and observed σ = 3.2, a sample size of 135 was required to detect the effect with 90% power. To account for early discontinuation, planned enrollment was 150–165 participants. For the adult trial, it was assumed that due to greater heterogeneity in the adult ADHD population, the TOVA‑ACS σ would be 1.5 times that observed with pediatric populations (STARS‑ADHD), where the standard deviation was 3.2. Assuming σ = 4.8 and the previously observed control-corrected effect size of µ = 0.9, a sample size of 301 participants was required to detect the effect with 90% power. To account for early discontinuation, planned enrollment was 325 participants.

#### Analysis populations

The trials yielded three analysis populations. Safety analyses were conducted on the Safety Population, which included all exposed to AKL-T01. The Efficacy Population consisted of all participants who took AKL-T01 home and completed both baseline and end of study assessments. The Per Protocol population was the subset of the Efficacy Population that met the definition for “adequate dosage” or “minimum acceptable exposure” to the game. For adolescents, this was defined as completion of at least 60% of missions, equivalent to 72 of 120 missions (at least 6 missions/day × 5 days/week × 4 weeks), and having the day 28 exit visit occur between day 22 and day 34, inclusive. For adults, this was defined as completion of at least 60% of missions, equivalent to 108 of 180 missions (at least 6 missions/day × 5 days/week × 6 weeks), and having the day 42 exit visit occur between day 36 and day 48, inclusive.

The primary endpoint was change in TOVA-ACS, calculated as the score at day 28 (adolescent) or day 42 (adult) minus the score at baseline for each participant. A one-sample t-test (0.05 significance level) was used to test the null hypothesis that change from baseline equals 0. The samples were of sufficient size such that the test was robust to deviations from the normality assumption due to the central limit theorem. We also conducted post-hoc sensitivity analyses for the primary outcome for each study using non-parametric Wilcoxon Signed Rank tests of change in TOVA-ACS from baseline to exit. A similar set of t-tests was conducted for secondary endpoints. The familywise type 1 error rate for the primary and secondary efficacy endpoints was 0.025 and was controlled using the fixed sequence method^29^. For responder analyses, responder rates were reported descriptively by number and percentage of patients meeting each response definition (see above). We also compared efficacy on primary and secondary endpoints across patients who were versus were not using concomitant stimulant medication. Note that while study participants could be taking other (i.e., non-stimulant) psychoactive medication, subgroup analyses are specifically based on concomitant *stimulant* use only.

Safety endpoints summarized for the Safety Population included incidence, severity, and relationship to study treatment of treatment-emergent adverse device effects (TE-ADEs) and were reported overall and by MedDRA system organ class (SOC) and preferred term (PT).

### Supplementary Information

Supplementary Methods

## Data Availability

The STARS-ADHD-Adolescents and STARS-ADHD-Adults Investigators agree to share de-identified individual participant data, the study protocol, and the statistical analysis plan with academic researchers 6 months after publication, and following completion of a Data Use Agreement. Proposals should be directed to medinfo@akiliinteractive.com.

## References

[1] Sibley MH, Mitchell JT, Becker SP (2016). Method of adult diagnosis influences estimated persistence of childhood ADHD: a systematic review of longitudinal studies. Lancet Psychiatry.

[2] Danielson M. L. Trends in Stimulant Prescription Fills Among Commercially Insured Children and Adults—United States, 2016–2021. *MMWR Morb Mortal Wkly Rep*. 2023;72. 10.15585/mmwr.mm7213a1. 10.15585/mmwr.mm7213a1 PMC10078845 36995976

[3] Blum D. Amid the Adderall Shortage, People With A.D.H.D. Face Withdrawal and Despair. *The New York Times*. https://www.nytimes.com/2022/11/16/well/mind/adderall-shortage-withdrawal-symptoms-adhd.html. Published November 16, 2022. Accessed May 25, 2023.

[4] What’s Driving the Demand for ADHD Drugs Like Adderall. Time. Published April 12, 2023. Accessed May 25, 2023. https://time.com/6271049/adhd-diagnoses-rising/.

[5] American Psychiatric Association. *Diagnostic and Statistical Manual of Mental Disorders (DSM-5®)*. American Psychiatric Publishing; 2013.

[6] Wolraich ML (2019). Clinical practice guideline for the diagnosis, evaluation, and treatment of attention-deficit/hyperactivity disorder in children and adolescents. Pediatrics.

[7] Biederman J (2015). Evidence of a pharmacological dissociation between the robust effects of methylphenidate on ADHD symptoms and weaker effects on working memory. J. Brain Sci..

[8] Mckenzie A (2022). The effects of psychostimulants on cognitive functions in individuals with attention-deficit hyperactivity disorder: a systematic review. J. Psychiatr. Res.

[9] Cortese S (2018). Comparative efficacy and tolerability of medications for attention-deficit hyperactivity disorder in children, adolescents, and adults: a systematic review and network meta-analysis. Lancet Psychiatry.

[10] Imboden AD, Fehr KK (2018). Collaborative care of attention deficit hyperactivity disorder: an innovative partnership to serve rural pediatric patients. J. Pediatr. Health Care.

[11] Baweja R, Soutullo CA, Waxmonsky JG (2021). Review of barriers and interventions to promote treatment engagement for pediatric attention deficit hyperactivity disorder care. World J. Psychiatry.

[12] Biederman J. et al. Evidence of low adherence to stimulant medication among children and youths with ADHD: an electronic health records study. *Psychiatr Serv*. 2safe019;70:874-880. 10.1176/appi.ps.201800515. 10.1176/appi.ps.201800515 31242830

[13] Brinkman WB, Simon JO, Epstein JN (2018). Reasons why children and adolescents with attention-deficit/hyperactivity disorder stop and restart taking medicine. Acad. Pediatr..

[14] Molina BSG (2009). The MTA at 8 years: prospective follow-up of children treated for combined-type ADHD in a multisite study. J. Am. Acad. Child Adolesc. Psychiatry.

[15] Visser SN (2014). Trends in the parent-report of health care provider-diagnosed and medicated attention-deficit/hyperactivity disorder: United States, 2003-2011. J. Am. Acad. Child Adolesc. Psychiatry.

[16] Faraone SV (2015). Attention-deficit/hyperactivity disorder. Nat. Rev. Dis. Prim..

[17] Döpfner M, Hautmann C, Görtz-Dorten A, Klasen F, Ravens-Sieberer U, BELLA study group. (2015). Long-term course of ADHD symptoms from childhood to early adulthood in a community sample. Eur. Child Adolesc. Psychiatry.

[18] Klingberg T (2010). Training and plasticity of working memory. Trends Cogn. Sci..

[19] Cortese S (2015). Cognitive training for attention-deficit/hyperactivity disorder: meta-analysis of clinical and neuropsychological outcomes from randomized controlled trials. J. Am. Acad. Child Adolesc. Psychiatry.

[20] Device Classification Under Section 513(f)(2)(De Novo). Accessed May 25, 2023. https://www.accessdata.fda.gov/scripts/cdrh/cfdocs/cfpmn/denovo.cfm?ID=DEN200026.

[21] Instructions For Use | EndeavorRx®. EndeavorRx. Accessed May 25, 2023. https://www.endeavorrx.com/instructions-for-use/.

[22] Leark R. A., Greenberg L. K., Kindschi C. L., Dupuy T. R., Hughes S. J. *Test of Variables of Attention: Professional Manual*. The TOVA Company.

[23] Kollins SH (2020). A novel digital intervention for actively reducing severity of paediatric ADHD (STARS-ADHD): a randomised controlled trial. Lancet Digit Health.

[24] Kollins SH, Childress A, Heusser AC, Lutz J (2021). Effectiveness of a digital therapeutic as adjunct to treatment with medication in pediatric ADHD. NPJ Digit Med.

[25] Larsson H, Dilshad R, Lichtenstein P, Barker ED (2011). Developmental trajectories of DSM-IV symptoms of attention-deficit/hyperactivity disorder: genetic effects, family risk and associated psychopathology. J. Child Psychol. Psychiatry.

[26] Rosenstock IM, Strecher VJ, Becker MH (1988). Social learning theory and the Health Belief Model. Health Educ. Q.

[27] Adler LA (2006). Validity of pilot adult ADHD self-report scale (ASRS) to rate adult ADHD symptoms. Ann. Clin. Psychiatry.

[28] Zhang S, Faries DE, Vowles M, Michelson D (2005). ADHD Rating Scale IV: psychometric properties from a multinational study as a clinician-administered instrument. Int J. Methods Psychiatr. Res..

[29] Hinshaw SP, Arnold LE (2015). For the MTA cooperative group. ADHD, multimodal treatment, and longitudinal outcome: evidence, paradox, and challenge. Wiley Interdiscip. Rev. Cogn. Sci..

[30] Nasser A (2022). A Phase III, randomized, double-blind, placebo-controlled trial assessing the efficacy and safety of viloxazine extended-release capsules in adults with attention-deficit/hyperactivity disorder. CNS Drugs.

[31] Adler LA (2022). Efficacy, safety, and tolerability of centanafadine sustained-release tablets in adults with attention-deficit/hyperactivity disorder. J. Clin. Psychopharmacol..

[32] Keefe RSE, Cañadas E, Farlow D, Etkin A (2022). Digital intervention for cognitive deficits in major depression: a randomized controlled trial to assess efficacy and safety in adults. Am. J. Psychiatry.

[33] Sheehan DV (2010). Reliability and validity of the mini international neuropsychiatric interview for children and adolescents (MINI-KID). J. Clin. Psychiatry.

[34] Walters SO, Weaver KA (2003). Relationships between the Kaufman brief intelligence test and the wechsler adult intelligence scale-third edition. Psychol. Rep..

[35] Raggio DJ, Scattone D, May W (2010). Relationship of the Kaufman brief intelligence test-second edition and the wechsler abbreviated scale of intelligence in children referred for ADHD. Psychol. Rep..

[36] Sheehan DV (1998). The Mini-International Neuropsychiatric Interview (M.I.N.I.): the development and validation of a structured diagnostic psychiatric interview for DSM-IV and ICD-10. J. Clin. Psychiatry.

[37] Greenberg L. K., Kindschi C. L., Dupuy T. R., Holder C. *Test of Variables of Attention: Clinical Manual*. The TOVA Company.

[38] DuPaul GJ, Power TJ, Anastopoulos AD, Reid R (2016). *ADHD Rating Scale-5 for Children and Adolescents: Checklists, Norms, and Clinical Interpretation*. Guilford Press.

[39] Brod M, Johnston J, Able S, Swindle R (2006). Validation of the adult attention-deficit/hyperactivity disorder quality-of-life scale (AAQoL): a disease-specific quality-of-life measure. Qual. Life Res..

[40] Conners C. K., Erhardt D., Sparrow E. P. *Conners’ Adult ADHD Rating Scales (CAARS): Technical Manual*. Multi-Health Systems North Tonawanda, NY; 1999.

