# Supplementary material for: Two single arm trials of AKL-T01, a digital therapeutic for adolescents and adults with ADHD
Source: Npj Ment Health Res. 2024 Jun 19;3:30. doi: 10.1038/s44184-024-00075-w (PMC11187123; doi:10.1038/s44184-024-00075-w)
Supplement: Supplementary file 1 — Supplementary Methods [file 44184_2024_75_MOESM1_ESM.docx]

**Supplementary Materials: Single-Arm Trial**

**Supplementary Methods**

***Single-arm trial***

As noted in the main text, the explicit intent of the single-arm studies detailed in this manuscript was to demonstrate substantial equivalence to an FDA-cleared predicate treatment for pediatric ADHD. To demonstrate substantial equivalence, there is explicit regulatory guidance in CFR 806.7(c)(2) supporting the use of single-arm studies as “valid scientific evidence.” There are several examples of industry precedents for using single-arm designs for this purpose: for instance, Freespira, which was initially FDA-cleared for use in panic disorder, employed a small single-arm trial (n=55) to achieve label expansion for a new indication, PTSD.^1^ Despite this industry precedent and explicit regulatory guidance, we nevertheless carefully considered a number of factors in making the decision to employ a single-arm study to evaluate AKL-T01 in adolescents and adults, as detailed below.

For both the adolescent and adult trials, we leveraged internal and external clinical data in making the decision to employ a single-arm trial design. The largest drawback to a single-arm trial is the possibility of a placebo effect. At the same time, unnecessarily assigning participants with a documented psychiatric condition to a sham condition comes with its own potential harms and ethical challenges.^2^ In weighing these considerations, we heavily considered the strong evidence of a lack of placebo effect on our primary outcome variable, TOVA. The TOVA is an FDA-cleared medical device for objectively assessing attention and aiding in the diagnosis of ADHD and treatment monitoring. Prior studies, both those evaluating AKL-T01 and those evaluating other ADHD treatments, support the lack of a placebo effect on the TOVA; this literature is summarized below.

*AKL-T01 and related treatments.* Three double-blind, parallel-group, randomized controlled trials (RCTs), have tested AKL-T01 and similar treatments against an expectation-matched digital placebo control on the TOVA assessment.^3–5^ The control used in each of these studies was a digital word game targeting cognitive domains not targeted by the AKL-T01 intervention. All 3 RCTs demonstrated a lack of placebo response on the TOVA composite score (a measure where positive changes indicate improvement), with effect sizes in the placebo groups ranging from -0.29 to 0.01:

- STARS-ADHD (Kollins 2020), the predicate study and largest of these trials, showed no material improvement in the placebo group in a pediatric sample.
- No improvement was observed in the placebo group of a small RCT of children with comorbid autism and ADHD (Yerys 2019). For this study, the placebo group showed deterioration on the TOVA.
- An RCT in adults with Major Depressive Disorder (MDD) also showed no improvement in the placebo group (Keefe 2022)—in this study, the placebo group again showed deterioration on the TOVA.

Supplementary Table 1 shows baseline and post-intervention mean change in the TOVA composite score in the STARS-ADHD-Adult and STARS-ADHD-Adolescent open-label trials and the placebo control groups from the three prior RCTs. The consistent lack of a placebo response on the TOVA composite score across these studies of AKL-T01, in both pediatric and adult samples, support the appropriateness of single-arm trials.

**Supplementary Table 1. Placebo response on the TOVA Composite Score (ACS) across studies of AKL-T01 and related treatments relative to treatment effects in the current studies**

|  | AKL-T01 | AKL-T01 | | Placebo | Placebo | | | Placebo |
| --- | --- | --- | --- | --- | --- | --- | --- | --- |
| Measure | STARS-ADHD-  Adolescent  N = 146 | | STARS-ADHD-  Adult  N = 153 | STARS-ADHD  RCT  N = 168 | | Autism+ADHD RCT  N = 7 | STARS-MDD  RCT  N = 39 | |
| Indication | ADHD | | ADHD | ADHD | | Comorbid Autism and ADHD | Major Depressive Disorder | |
| Age Range | 13-17y | | 18-76y | 8-12y | | 9-13y | 25-55y | |
| Baseline TOVA Composite Score* |  | |  |  | |  |  | |
| N evaluated | 146 | | 153 | 168 | | 7 | 39 | |
| Mean (SD) | -5.447 (3.753) | | -8.739 (7.532) | -4.936 (3.083) | | -4.628 (2.809) | -2.410 (6.279) | |
| Change from Baseline |  | |  |  | |  |  | |
| N evaluated | 146 | | 153 | 160 | | 7 | 37 | |
| Mean (SD) | 2.639 (3.799) | | 6.460 (6.952) | 0.028 (3.164) | | -0.826 (3.405) | -1.400 (4.427) | |
| 95% CI | 2.018, 3.261 | | 5.349, 7.570 | -0.466, 0.522 | | -3.975, 2.324 | -2.876, 0.076 | |
| P-value | <0.0001 | | <0.0001 | 0.91 | | 0.54 | 0.06 | |
| Effect size | 0.70 | | 0.86 | 0.01 | | -0.29 | -0.22 | |

*External literature supporting lack of a placebo effect on TOVA.* Outside of previous studies of AKL-T01 and related treatments, there are numerous external studies substantiating a lack of placebo effect on TOVA outcomes. We identified nine published studies that met the criteria of being double-blind, placebo controlled RCTs using the TOVA as an outcome in children, adolescents, and adults with ADHD.^6–15^ Among studies with pre- and post-treatment measures available to calculate placebo response for TOVA-ACS, effect sizes for the change in TOVA-ACS in the placebo condition were small, ranging from -0.05 to 0.19 in pediatric and adolescent studies,^6–13^ with a maximum of a small effect (.38) in one adult study.^14,15^

**Sensitivity Analyses**

While the external literature has consistently demonstrated no or minimal effect of placebo on the TOVA among pediatric and adolescent populations, the possibility of a small placebo response for adults on TOVA led us to a sensitivity analysis in our adult population using estimates of placebo response for TOVA-ACS found in the external literature. The most pronounced placebo effect for this measure identified in the literature was an RCT of metadoxine in adults aged 18 to 50 years with primarily inattentive- or combined-type ADHD.^14^ The 60 participants in the placebo group of this trial improved from a baseline mean (SD) TOVA-ACS of -7.3 (7.2) to -4.2 (5.6) after 6 weeks, corresponding to a mean (SD) change of 3.0 (6.4). The similar patient population (in age, diagnosis, and baseline attentional impairment) and identical duration of intervention (6 weeks) supported use of this estimate as a performance goal to benchmark efficacy of AKL-T01 accounting for placebo effects. The analysis used a one-sample t-test to compare the mean change in TOVA-ACS in our adult study to a mean change of 3.0 as a performance goal based on this external study.

**Supplementary Results**

**Sensitivity Analyses**

Results from our sensitivity analyses are shown in Supplementary Table 2 and display the statistical comparison of the observed mean change in TOVA-ACS for adults relative to placebo estimate (performance goal of 3.0) from the existing scientific literature. The analysis supports that even if we had included a control group, the observed change in TOVA-ACS would have surpassed high estimates of a placebo effect (p<.0001).

**Supplementary Table 2. Sensitivity Analysis of Primary Efficacy Endpoint - TOVA-ACS Change from Baseline to Exit in the Efficacy Population**

| Measure | Baseline | Exit | Change from Baseline |
| --- | --- | --- | --- |
| TOVA-ACS in Adults 18y+ |  |  |  |
| N evaluated | 153 | 153 | 153 |
| Mean (SD) | -8.74 (7.53) | -2.28 (4.92) | 6.46 (6.95) |
| 2-Sided 95% CI^1^ |  |  | 5.35, 7.57 |
| P-value: PG = 3.0^2^ |  |  | <0.0001 |

Abbreviations: TOVA-ACS, Test of Variables of Attention-Attention Comparison Score; N, number of subjects in population; SD, standard deviation; min, minimum; max, maximum; CI, confidence interval; PG, performance goal.

^1^ Positive change indicates improvement. Efficacy is supported if the lower bound of the CI is greater than PG.

^2^ From a one-sample t-test with H_0_ = 0.

^3^ From a one-sample t-test with H_0_ = 3.0.

**Supplementary Discussion**

Taken together, these findings support the use of a single-arm trial. For one, there is substantial documentation in the scientific literature that demonstrates the lack of placebo effect on TOVA outcomes. Further, for a placebo effect to be present in sufficient magnitude to influence evaluation of findings, the magnitude of such a placebo effect would be inconsistent with scientific understanding of placebo effects for TOVA documented in the literature. For instance, if a control condition had been included in the adolescent study, we could have observed as high as 1.7-point TOVA ACS improvement (corresponding to a placebo group effect size of 0.5) and still had sufficient statistical power to detect a significant between group effect of our treatment. This placebo effect would have been approximately twice as large as the largest observed placebo effect in any of the published pediatric or adolescent ADHD RCTs summarized above.

**Supplementary Materials: Poolability**

**Supplementary Methods**

***Poolability Across Sites***

We conducted post-hoc analyses to evaluate potential differences across sites, and to determine if pooling results across sites was appropriate.

**Supplementary Results**

***Adolescent Trial***

No significant differences by site were detected (*p*=0.57; Supplementary Table 3). Results stratified by site showed consistent improvement in TOVA-ACS, ranging from a minimum mean change (SD) in TOVA-ACS of 1.318 (3.531) at Site 12 (N=6) to a maximum of 5.418 (5.339) at Site 2 (N=10). Given the common protocol across study sites and observed homogeneity of effect, site pooling is justified.

**Supplementary Table 3. Adolescent Trial: Primary Outcome Stratified by Study Site – Change in TOVA-ACS from Baseline**

| Study Site | N | Mean Change (SD) in TOVA-ACS from Baseline |
| --- | --- | --- |
| All Sites | 146 | 2.639 (3.799) |
| Site 03 | 23 | 2.218 (4.124) |
| Site 07 | 22 | 2.743 (2.887) |
| Site 06 | 19 | 3.352 (4.848) |
| Site 08 | 15 | 2.613 (3.826) |
| Site 05 | 11 | 3.283 (3.337) |
| Site 10 | 11 | 1.331 (2.714) |
| Site 02 | 10 | 5.418 (5.339) |
| Site 09 | 10 | 2.248 (3.399) |
| Site 01 | 9 | 1.717 (3.527) |
| Site 12 | 6 | 1.318 (3.531) |
| Site 11 | 5 | 1.640 (3.127) |
| Site 04 or 14 | 5 | 2.430 (2.699) |
| p-value for difference by site | p = 0.57 | |

***Adult Trial***

Again, no significant differences in the primary efficacy endpoint, change in TOVA-ACS from baseline to study day 42, were observed when stratified by study site (*p* = 0.24; Supplementary Table 4). Given the common protocol across study sites and observed homogeneity of effect, site pooling is justified.

**Supplementary Table 4. Adult Trial: Primary Efficacy Endpoint Stratified by Study Site - TOVA-ACS Change from Baseline**

| Study Site | N | Mean Change (SD) in TOVA-ACS from Baseline |
| --- | --- | --- |
| All Sites | 153 | 6.460 (6.952) |
| Site 10 | 37 | 6.129 (7.548) |
| Site 07 | 28 | 5.679 (4.252) |
| Site 08 | 17 | 6.286 (7.520) |
| Site 12 | 17 | 4.082 (4.872) |
| Site 02 | 13 | 9.733 (8.135) |
| Site 06 | 8 | 8.663 (6.985) |
| Site 01 | 7 | 4.261 (1.405) |
| Site 11 | 7 | 3.796 (5.731) |
| Site 14 | 6 | 7.125 (7.013) |
| Site 04, 05, 09, or 131 | 13 | 10.103 (10.717) |
| p-value for differences by site | 0.24 | |

*Abbreviations: TOVA-ACS, Test of Variables of Attention-Attention Comparison Score; N, number of subjects in population; SD, standard deviation.*

*^1^ Sites with 5 or fewer participants were combined to ensure more stable estimates. These sites were 04 (N=2), 05 (N=1), 09 (N=5), and 13 (N=5).*

**Supplementary Materials: Percent Compliance by Study Week**

**Supplementary Table 5. Average Percent Compliance by Study Week (Adolescent Study)**

|  | Percent Compliance *M (SD)* | | |
| --- | --- | --- | --- |
| Study Week | Safety  Population   (N=162) | Efficacy  Population   (N=146) | Per Protocol  Population   (N=74) |
| 1 | 81.07 (46.867) | 86.55 (45.572) | 113.74 (39.769) |
| 2 | 63.52 (48.758) | 69.27 (47.478) | 98.51 (39.866) |
| 3 | 55.82 (46.484) | 61.51 (45.395) | 91.17 (36.245) |
| 4 | 56.73 (51.235) | 62.69 (50.467) | 89.77 (45.032) |

**Supplementary Table 6. Average Percent Compliance by Study Week (Adult Study)**

|  | Percent Compliance *M (SD)* | | |
| --- | --- | --- | --- |
| Study Week | Safety  Population   (N=162) | Efficacy  Population   (N=146) | Per Protocol  Population   (N=74) |
| 1 | 83.83 (50.814) | 98.80 (47.092) | 117.36 (41.774) |
| 2 | 70.32 (54.550) | 88.22 (51.196) | 115.91 (38.184) |
| 3 | 63.98 (58.235) | 83.46 (55.972) | 114.03 (41.237) |
| 4 | 56.83 (58.611) | 75.75 (56.901) | 106.60 (46.680) |
| 5 | 50.57 (54.644) | 69.76 (54.451) | 98.22 (43.866) |
| 6 | 51.18 (63.109) | 70.55 (65.035) | 100.66 (58.600) |

**Supplementary Materials: Detailed Inclusion and Exclusion Criteria**

**Supplementary Table 7. Adolescent Study Inclusion and Exclusion Criteria**

| **Inclusion Criteria** |
| --- |
| 1. Adolescents between the ages of 13 and 17 years and 10 months at time of consent (must be under 18 years at study completion) |
| 1. Confirmed diagnosis of ADHD combined or inattentive type, according to Diagnostic and Statistical Manual of Mental Disorders, Fifth Edition (DSM-5) as confirmed by MINI-Kid Version 7.0.2. |
| 1. Stably on or off ADHD medication for ≥4 weeks prior to study enrollment and throughout the 4-week study |
| 1. Baseline visit score on the TOVA-ACS score ≤ -1.8 |
| 1. Access to and self-report of ability to connect wireless devices to a functional wireless network |
| 1. Ability to follow written and verbal instructions (English) as assessed by the PI and/or study coordinator |
| 1. Able to comply with all testing and study requirements |
| 1. Estimated IQ score ≥80 as assessed by the Kaufmann Brief Intelligence Test, Second Edition (KBIT-II) |
| 1. Patient assent and caregiver informed consent |
| 1. Stably on or off psychoactive medications for ≥ 4 weeks prior to study enrollment and throughout the 4-week study |
| **Exclusion Criteria** |
| 1. Current controlled or uncontrolled, comorbid psychiatric diagnosis that in the opinion of the Investigator may confound study data/assessments. |
| 1. Participant is currently considered at risk for attempting suicide, has made a suicide attempt within the past year, or is currently demonstrating active suicidal ideation or self-injurious behavior, in the opinion of the Investigator based on the MINI-kid clinical interview. |
| 1. Motor condition (e.g., physical deformity of the hands/arms) that prevents game playing as reported by the participant or observed by the Investigator. |
| 1. Recent history (6 months prior to screening) of substance use disorder |
| 1. History of seizures (excluding febrile seizures), significant tics, or a current diagnosis of Tourette’s Disorder. |
| 1. Known sensitivity to playing video games, such as photo- sensitive epilepsy, light-headedness, dizziness, nausea, or motion sickness. |
| 1. Participation in a clinical trial within 3 months prior to screening. |
| 1. Plans to initiate, or to make significant changes in frequency, of non-pharmacological behavioral therapy during the study |
| 1. Color blindness as detected by Ishihara Color Blindness Test |
| 1. Urine test positive for nicotine or marijuana |
| 1. Any other medical condition that in the opinion of the Investigator may confound study data/assessments. |
| 1. Previous exposure to Akili products within the 6 months prior to study enrollment |
| 1. Plans to initiate or to make significant changes in frequency or duration of non-pharmacological trainings with the aim to improve cognition by means of game or app-based cognitive trainings or neurofeedback, during the study |

**Supplementary Table 8. Adult Study Inclusion and Exclusion Criteria**

| **Inclusion Criteria** |
| --- |
| 1. Adults 18 years and older |
| 1. Diagnosis of ADHD combined or inattentive type, according to Diagnostic and Statistical Manual of Mental Disorders, Fifth Edition (DSM-5) as confirmed by Mini International Neuropsychiatric Interview (MINI) for Attention – Deficit / Hyperactivity Disorders Studies (Adult) 7.0.2 |
| 1. Stably on or off ADHD medications for ≥4 weeks prior to study enrollment and throughout the primary 6-week study |
| 1. Stably on or off psychoactive medications for ≥4 weeks prior to study enrollment and throughout the 4-week study |
| 1. Baseline visit score on the ADHD-RS-IV of ≥ 24 |
| 1. Baseline visit score on the TOVA-ACS score ≤ -1.8 |
| 1. Estimated IQ score ≥80 as assessed by the Kaufmann Brief Intelligence Test, Second Edition (KBIT-II) |
| 1. Access to and self-report of ability to connect wireless devices to a functional wireless network |
| 1. Ability to follow written and verbal instructions (English) as assessed by the PI and/or study coordinator |
| 1. Able to comply with all testing and study requirements |
| 1. Completion of informed consent form |
| **Exclusion Criteria** |
| 1. Current controlled or uncontrolled, comorbid psychiatric diagnosis with significant symptoms that in the opinion of the Investigator may confound study data/assessments. |
| 1. Suicidality assessed using the Columbia-Suicide Severity Rating Scale (C-SSRS) |
| 1. Motor condition (e.g., physical deformity of the hands/arms) that prevents game playing as reported by the participant or observed by the Investigator. |
| 1. History of moderate or severe substance use disorder within the last 12 months prior to informed consent |
| 1. History of seizures (excluding febrile seizures), significant tics, or a current diagnosis of Tourette’s Disorder. |
| 1. Known sensitivity to playing video games, such as photo-sensitive epilepsy, light-headedness, dizziness, nausea, or motion sickness. |
| 1. Color blindness as detected by Ishihara Color Blindness Test |
| 1. Positive urine drug screen |
| 1. Current or recent (3 months prior to screening) history of heavy smoking defined as the equivalent of greater than or equal to a pack of cigarettes a day |
| 1. Any other medical condition that in the opinion of the Investigator may confound study data/assessments. |
| 1. Participation in a clinical trial within 3 months prior to screening. |
| 1. Previous exposure to Akili products within the 6 months prior to study enrollment |
| 1. Plans to initiate new concomitant medications during the primary study, except for common over the counter (OTC) (e.g., ibuprofen, acetaminophen) and prescription medications (e.g., antibiotics) for minor transient ailments. |
| 1. Planned initiation of, or significant changes in frequency, of non-pharmacological behavioral therapy during the primary study |
| 1. Planned initiation of, or significant changes in frequency of, non-pharmacological trainings with the aim to improve cognition by means of game or app-based cognitive trainings or neurofeedback, during the primary study |

**Supplementary References**

1. Ostacher MJ, Fischer E, Bowen ER, Lyu J, Robbins DJ, Suppes T. Investigation of a Capnometry Guided Respiratory Intervention in the Treatment of Posttraumatic Stress Disorder. *Appl Psychophysiol Biofeedback*. 2021;46(4):367-376. doi:10.1007/s10484-021-09521-3

2. Ovosi JO, Ibrahim MS, Bello-Ovosi BO. Randomized controlled trials: Ethical and scientific issues in the choice of placebo or active control. *Ann Afr Med*. 2017;16(3):97-100. doi:10.4103/aam.aam_211_16

3. Kollins SH, DeLoss DJ, Cañadas E, et al. A novel digital intervention for actively reducing severity of paediatric ADHD (STARS-ADHD): a randomised controlled trial. *Lancet Digit Health*. 2020;2(4):e168-e178. doi:10.1016/S2589-7500(20)30017-0

4. Yerys BE, Bertollo JR, Kenworthy L, et al. Brief Report: Pilot Study of a Novel Interactive Digital Treatment to Improve Cognitive Control in Children with Autism Spectrum Disorder and Co-occurring ADHD Symptoms. *J Autism Dev Disord*. 2019;49(4):1727-1737. doi:10.1007/s10803-018-3856-7

5. Keefe RSE, Cañadas E, Farlow D, Etkin A. Digital Intervention for Cognitive Deficits in Major Depression: A Randomized Controlled Trial to Assess Efficacy and Safety in Adults. *Am J Psychiatry*. 2022;179(7):482-489. doi:10.1176/appi.ajp.21020125

6. González-Castro P, Cueli M, Rodríguez C, García T, Álvarez L. Efficacy of Neurofeedback Versus Pharmacological Support in Subjects with ADHD. *Appl Psychophysiol Biofeedback*. 2016;41(1):17-25. doi:10.1007/s10484-015-9299-4

7. Katz M, Levine AA, Kol-Degani H, Kav-Venaki L. A compound herbal preparation (CHP) in the treatment of children with ADHD: a randomized controlled trial. *J Atten Disord*. 2010;14(3):281-291. doi:10.1177/1087054709356388

8. Murray DW, Childress A, Giblin J, Williamson D, Armstrong R, Starr HL. Effects of OROS methylphenidate on academic, behavioral, and cognitive tasks in children 9 to 12 years of age with attention-deficit/hyperactivity disorder. *Clin Pediatr (Phila)*. 2011;50(4):308-320. doi:10.1177/0009922810394832

9. Raz R, Carasso RL, Yehuda S. The influence of short-chain essential fatty acids on children with attention-deficit/hyperactivity disorder: a double-blind placebo-controlled study. *J Child Adolesc Psychopharmacol*. 2009;19(2):167-177. doi:10.1089/cap.2008.070

10. Rugino TA, Samsock TC. Modafinil in children with attention-deficit hyperactivity disorder. *Pediatr Neurol*. 2003;29(2):136-142. doi:10.1016/s0887-8994(03)00148-6

11. Vaisman N, Kaysar N, Zaruk-Adasha Y, et al. Correlation between changes in blood fatty acid composition and visual sustained attention performance in children with inattention: effect of dietary n-3 fatty acids containing phospholipids. *Am J Clin Nutr*. 2008;87(5):1170-1180. doi:10.1093/ajcn/87.5.1170

12. Voigt RG, Llorente AM, Jensen CL, Fraley JK, Berretta MC, Heird WC. A randomized, double-blind, placebo-controlled trial of docosahexaenoic acid supplementation in children with attention-deficit/hyperactivity disorder. *J Pediatr*. 2001;139(2):189-196. doi:10.1067/mpd.2001.116050

13. Wigal SB, Wigal T, Schuck S, et al. Academic, Behavioral, and Cognitive Effects of OROS® Methylphenidate on Older Children with Attention-Deficit/Hyperactivity Disorder. *J Child Adolesc Psychopharmacol*. 2011;21(2):121-131. doi:10.1089/cap.2010.0047

14. Manor I, Ben-Hayun R, Aharon-Peretz J, et al. A randomized, double-blind, placebo-controlled, multicenter study evaluating the efficacy, safety, and tolerability of extended-release metadoxine in adults with attention-deficit/hyperactivity disorder. *J Clin Psychiatry*. 2012;73(12):1517-1523. doi:10.4088/JCP.12m07767

15. Rotem A, Ben-Sheetrit J, Newcorn J, et al. The Placebo Response in Adult ADHD as Objectively Assessed by the TOVA Continuous Performance Test. *J Atten Disord*. 2021;25(9):1311-1320. doi:10.1177/1087054719897819
